# Intrauterine inflammation exposure may increase the risk of late-onset sepsis in premature infants: a retrospective cohort study

**DOI:** 10.1186/s13052-025-02040-5

**Published:** 2025-07-10

**Authors:** Xiafang Chen, XinYu Zhang, Ru Xue, Lanlan Mi, Ye Liu, Guoqing Zhang, Jun Bu, Fei Bei

**Affiliations:** 1 Department of Neonatology, Shanghai Children’s Medical Center, Shanghai Jiao Tong University School of Medicine, 1678 DongFang Road, Shanghai, 200127 China; 2 Department of Obstetrics and Gynecology, Renji Hospital, School of Medicine, Shanghai Jiao Tong University, Shanghai, China

**Keywords:** Late-onset sepsis, Preterm infants, Cytokines, Intrauterine inflammation

## Abstract

**Background:**

Preterm birth associated with intrauterine inflammation (IUI) has been linked to alterations in postnatal immunity and severe inflammatory complications during infancy. However, the impact of IUI on late-onset sepsis (LOS), a leading cause of mortality and morbidity in preterm infants, remains unclear. This study aims to elucidate the effect of IUI on the incidence of LOS in preterm infants by analyzing cytokine levels and white blood cell differential counts in cord blood within 24 h after birth.

**Methods:**

This retrospective cohort study was conducted at a single tertiary neonatal center. Infants born before 37 weeks of gestation between July 2020 and June 2022 were included. Late-onset sepsis (LOS) was defined as sepsis occurring after 72 h of life during the birth hospitalization. Levels of 12 cytokines, including interleukin-1β (IL-1β), IL-2, IL-4, IL-5, IL-6, IL-8, IL-10, IL-12p70, IL-17, tumor necrosis factor-α (TNF-α), interferon-α (IFN-α), and IFN-γ, were measured in cord blood using multiplex bead-based flow immunoassays. Clinical data were extracted from hospital databases. Peripheral white blood cell counts within 24 h after birth were routinely recorded for preterm infants. Logistic regression analysis was used to assess the impact of cytokines and white blood cell counts on the incidence of LOS.

**Results:**

A total of 628 preterm infants were included in this study. The mean gestational age was 33.17 ± 2.25 weeks, and the mean birth weight was 1929.50 ± 516.77 g. Of these, 42 infants (6.7%) developed late-onset sepsis (LOS). Compared to the non-LOS group, cord blood levels of IL-6 [127.81 (399.86) vs. 31.02 (127.48), *p* = 0.004] and IL-8 [130.37 (202.53) vs. 52.91 (101.43), *p* = 0.001] were significantly higher in the LOS group. No significant differences were observed in the levels of other cytokines between the groups. Peripheral neutrophil and monocyte counts were significantly lower in the LOS group [5.08 ± 3.46 vs. 8.14 ± 4.90, *p* < 0.001; 0.98 ± 0.56 vs. 1.37 ± 0.72, *p* = 0.001, respectively]. Multivariable logistic regression analysis revealed that elevated cord blood IL-6 levels and reduced peripheral neutrophil counts were associated with an increased risk of LOS, after adjusting for gestational age, gestational hypertension, and antenatal steroid use (aOR = 3.113, 95% CI: 1.239–7.819, *p* = 0.016; aOR = 0.340, 95% CI: 0.818–0.994, *p* = 0.038, respectively).

**Conclusions:**

Elevated cord blood IL-6 levels and low peripheral neutrophil counts on the first day after birth are associated with an increased risk of LOS in preterm infants. These findings highlight the potential of these non-invasive biomarkers in clinical practice to improve the prediction of LOS risk. Early identification using these markers may facilitate targeted management strategies, thereby reducing complications and mortality rates. Moreover, the association suggests that intrauterine inflammation may have a lasting impact on immune system responses, potentially influencing susceptibility to infections later in life.

## Background

Late-onset sepsis (LOS), defined as sepsis occurring after 72 h of life during the birth hospitalization, is a major cause of morbidity and mortality among preterm infants, with reported incidence rates ranging from 20 to 30% among the most frequently hospitalized preterm infants [1]. Infants who develop LOS are at increased risk of short-term in-hospital complications, mortality, and adverse long-term neurodevelopmental outcomes at 2 years of corrected age among survivors [2, 3]. Despite numerous prevention strategies implemented over the past decade, outcomes for preterm infants with LOS have remained unchanged.

Intrauterine infection/inflammation (IUI) is a significant etiological factor for preterm birth and is associated with conditions such as chorioamnionitis, maternal infection, and premature rupture of membranes (PROM). The concept of chorioamnionitis, also known as intrauterine inflammation or infection (commonly referred to as ‘Triple I’), encompasses both clinical and histological criteria [4].

Evidence suggests a complex relationship between intrauterine inflammation and preterm delivery, as well as the development of LOS [5–7]. While some studies have shown that intrauterine inflammation increases the incidence of LOS in preterm infants less than 28 weeks’ gestation, others have reported no significant association or even a reverse relationship [8, 9].

These discrepancies may be attributed to variations in the timing and duration of intrauterine inflammation. Infection without a corresponding fetal or maternal inflammatory response may not lead to preterm delivery. As the inflammatory process progresses, both pro-inflammatory cytokines (e.g., IL-1β, IL-4, IL-6, IL-8) and anti-inflammatory cytokines (e.g., IL-10) are produced. Given that pro-inflammatory cytokines rarely cross the placenta [10], cord blood cytokine levels can accurately reflect intrauterine inflammation, while peripheral white blood cell counts provide insight into the newborn’s immune status.

Assessing the relationship between intrauterine inflammation and LOS is crucial for enhancing our understanding of the innate immune response in preterm infants. This knowledge could inform the development of novel strategies for preventing and managing LOS. Therefore, this study aims to elucidate the association between intrauterine inflammation exposure and LOS by analyzing venous cord blood levels of IL-1β, IL-2, IL-4, IL-5, IL-6, IL-8, IL-10, IL-12p70, IL-17, TNF-α, IFN-α, IFN-γ, and counts of neutrophils, lymphocytes, and monocytes in a cohort of preterm neonates.

## Method

### Study design, setting, and participants

This retrospective cohort study was conducted in the Department of Neonatology at Shanghai Children’s Medical Center, Shanghai, China. We included preterm infants admitted to our center from July 2020 to June 2022. The exclusion criteria were: absence of cord blood samples, death within 72 h of birth, early-onset sepsis, and major congenital anomalies. Data were collected by trained data abstractors at Shanghai Children’s Medical Center. Late-onset sepsis (LOS) was defined as a positive blood or cerebrospinal fluid culture occurring after 72 h of life, excluding cases of specimen contamination. Gestational hypertension was defined as a blood pressure reading greater than 140 mm Hg systolic or greater than 90 mm Hg diastolic, measured on at least two separate occasions. Gestational diabetes was defined as any degree of glucose intolerance with onset or first recognition during pregnancy. Small for gestational age (SGA) was defined as infants with a weight below the 10th percentile of the Fenton growth curve for their gestational age. Preterm premature rupture of membranes (PPROM) was defined as the rupture of membranes occurring before 37 weeks of gestation. Antenatal steroids were routinely administered to mothers at risk of preterm delivery between 24 and 34 weeks gestational age. Although Group B Streptococcus (GBS) colonization in pregnancy is crucial in contributing to IUI [11] and GBS screening is a standard practice in pregnancy, our cohort did not include data on vaginal or rectal swabs for GBS colonization. This is primarily because GBS screening is typically conducted between 35 and 37 weeks of gestation, and most of our study population were born before 35 weeks.

### Exposure

We measured the serum concentrations of various cytokines (IL-1β, IL-2, IL-4, IL-5, IL-6, IL-8, IL-10, IL-12p70, IL-17, TNF-α, IFN-α, IFN-γ) from 2 mL cord blood samples collected from all participants. The cytokine concentrations were assessed using MBFFI test kits (MAGPIX^®^, USA). Additionally, white blood cell counts, including neutrophils, lymphocytes, and monocytes, were routinely performed in preterm infants within 24 h after birth.

### Statistical analysis

Continuous variables were expressed as mean and standard deviation (SD) or median and interquartile range (IQR; [Q1, Q3]) as appropriate, while categorical variables were presented as number and percentage. Differences between patients and controls were analyzed using Student’s t-test for normally distributed values, and the Mann–Whitney U-test for non-normally distributed data. Categorical variables were compared using the Chi-square test or Fisher’s exact test. Spearman correlation analysis was employed to evaluate the relationships between cytokines and clinical parameters. Multivariable logistic regression was utilized to assess the association of intrauterine inflammation with late-onset sepsis, adjusting for gestational age, gestational hypertension, and antenatal steroid use. All statistical analyses were conducted using IBM SPSS Statistics version 26.0 (IBM, Armonk, NY, USA), with a significance level set at *P* < 0.05 (two-sided).

### Ethics statement

The study protocol was reviewed and approved by the Ethics Board of Shanghai Children’s Medical Center, School of Medicine, Shanghai Jiao Tong University (SCMCIRB-K2021016-1). Written informed consent was obtained from all participants’ legal guardians, and approval to access clinical data was granted by the hospital’s medical director’s office. All patient information was kept confidential.

## Results

**Fig. 1:**
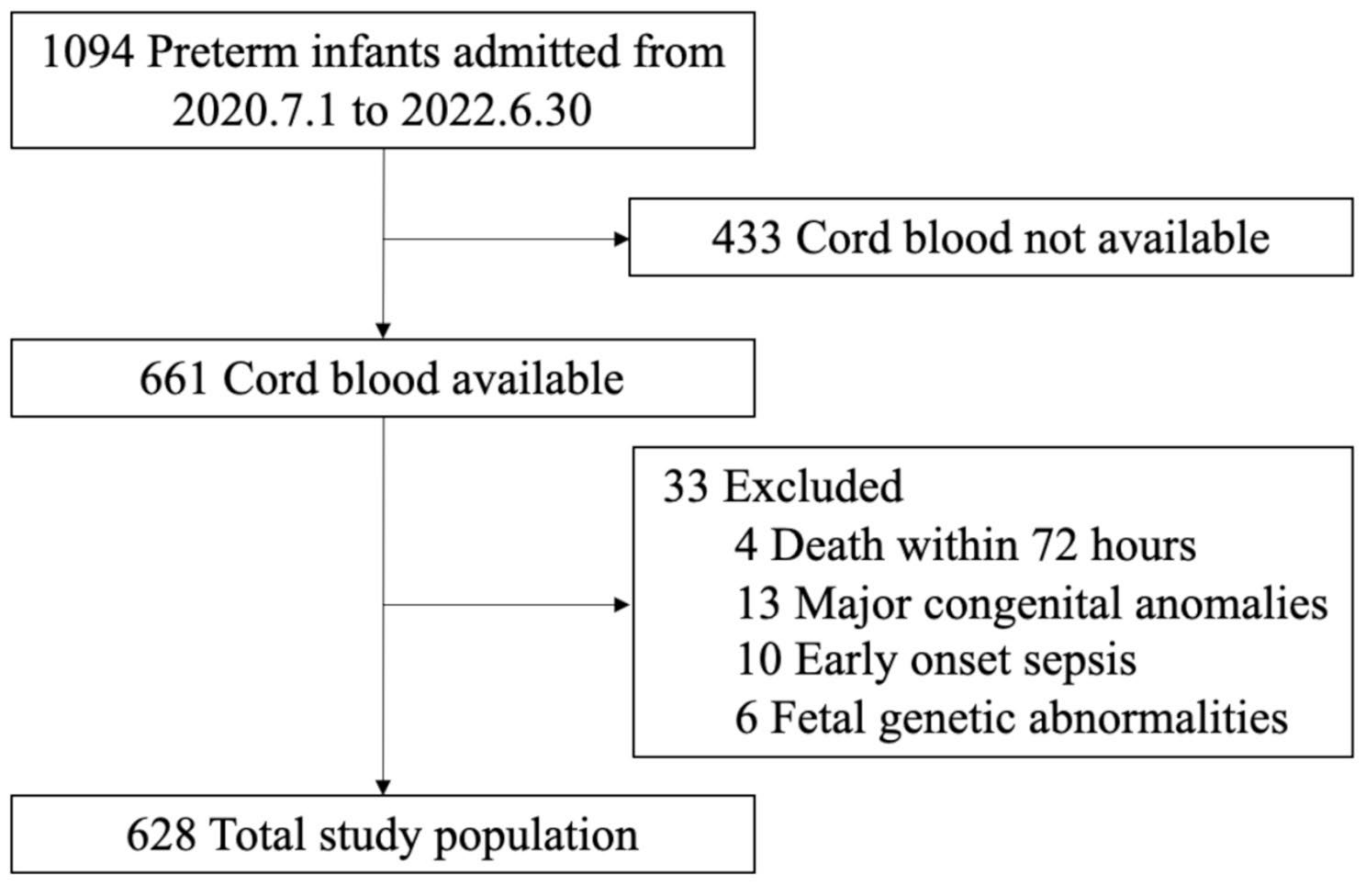
Flow diagram of study participants. The diagram illustrates the selection process and exclusion criteria applied to the cohort of preterm infants in the study. It highlights the initial pool of eligible participants, exclusion due to lack of cord blood samples, neonatal deaths within 72 h, early-onset sepsis, and major congenital anomalies, culminating in the final cohort analysis

**Fig. 2:**
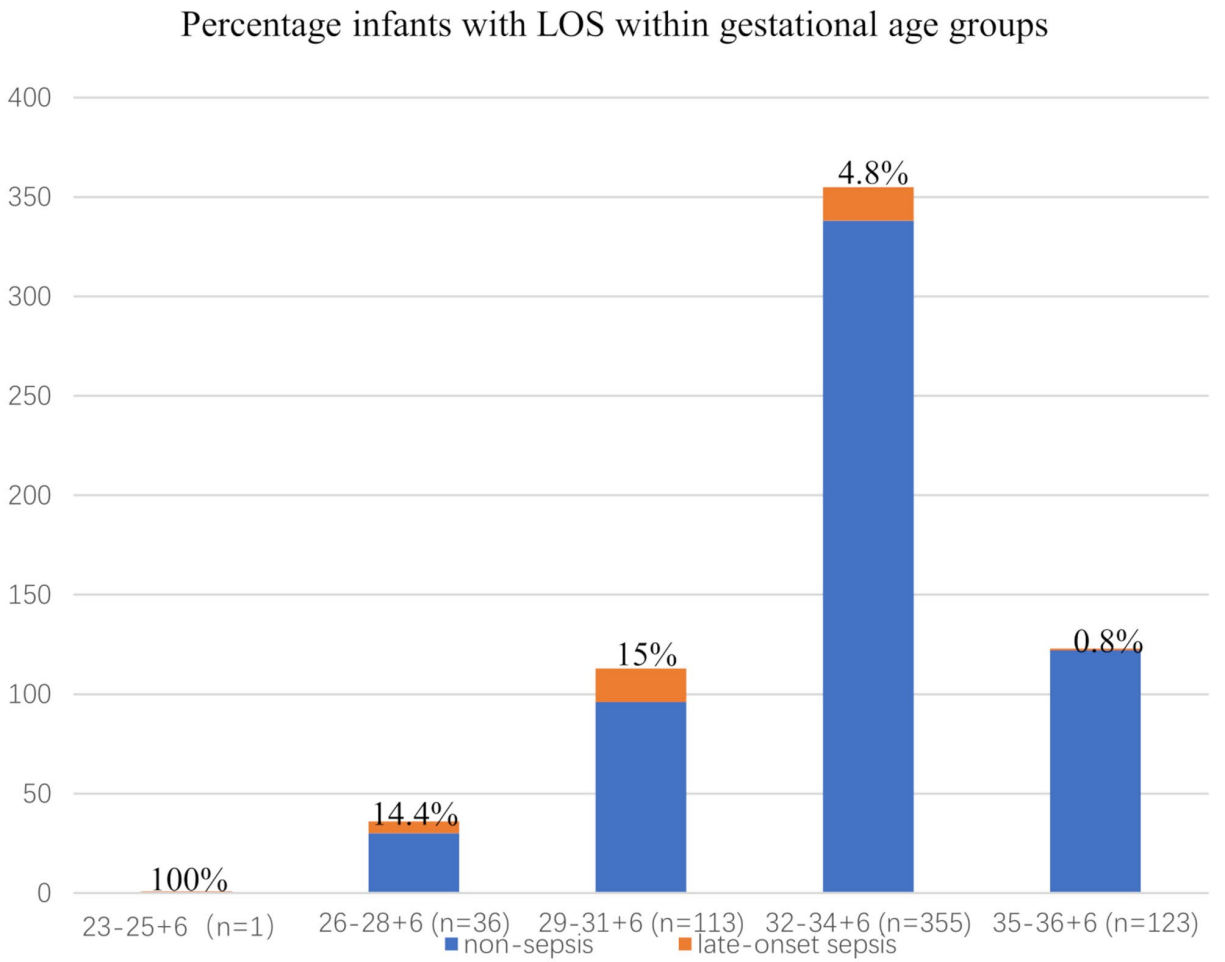
Gestational age distribution and percentage infants with LOS within gestational age groups. This figure illustrates the distribution of gestational ages among the study population and the corresponding percentage of infants diagnosed with late-onset sepsis (LOS) in each gestational age group. Blue bars represent the percentage of infants without sepsis, while yellow bars indicate the percentage of infants with LOS in each group

A total of 1,094 preterm neonates were admitted to our center from July 1, 2020, to June 30, 2022. Due to the unavailability of cord blood samples—either because of transfers from other hospitals or emergency deliveries—433 infants were excluded from the study. Additionally, 33 infants were excluded due to death within 72 h, major congenital abnormalities, early-onset sepsis, and fetal genetic abnormalities. Consequently, 628 infants were included in the analysis (Fig. 1). The mean gestational age (GA) of the cohort was 33.17 ± 2.25 weeks, and the mean birth weight (BW) was 1929.50 ± 516.77 g (Table 1). During the study period, 42 preterm infants developed late-onset sepsis (LOS), with a mean onset age of 15.69 days. The majority of infected infants were born before 32 weeks of gestation, representing 57% of cases. Notably, there was a positive correlation between gestational age and the incidence of LOS, indicating that a lower gestational age was associated with a higher occurrence of LOS (Fig. 2). The gestational age and birth weight of the LOS group were significantly lower compared to the non-sepsis group [31.26 ± 2.41 vs. 33.30 ± 2.18, *p* = 0.000; 1507.02 ± 498.27 vs. 1959.78 ± 505.07, *p* = 0.000, respectively]. Except for maternal hypertension and antenatal steroid use, there were no significant differences between the two groups regarding small for gestational age (SGA), sex, delivery mode, preterm premature rupture of membranes (PPROM), 1- and 5-minute Apgar scores, and gestational diabetes (Table 1).

**Table 1:** Demographic and clinical characteristics of the study population

	LOS group (*n* = 42)	Non-sepsis group (*n* = 586)	Total	*p*
Gestational age(wks)	31.26 ± 2.41	33.30 ± 2.18	33.17 ± 2.25	0.000**
Birth weight(g)	1507.02 ± 498.27	1959.78 ± 505.07	1929.50 ± 516.77	0.000**
Age of onset (days)	15.69 ± 8.23	/	/	/
SGA	7 (16.7%)	61 (10.5%)	68 (10.8%)	0.316
Gender, male	23(54.8%)	316(53.9%)	339(54.0%)	1.000
Cesarean	32(76.2%)	487(83.1%)	519(82.6%)	0.351
1 min Apgar ≤ 7	74 (14.6%)	7 (16.7%)	81 (12.9%)	0.609
5 min Apgar ≤ 7	18 (3.1%)	3 (7.1%)	21 (3.3%)	0.316
PPROM	8(19.0%)	76(13.0%)	84(13.4%)	0.377
Gestational hypertension, n (%)	13 (31.0%)	92 (15.7%)	105 (16.7%)	0.019*
Gestational diabetes, n (%)	12 (28.6%)	114 (19.5%)	126 (20.1%)	0.220
Antenatal steroid, n (%)	37 (88.1%)	398 (67.9%)	435 (69.3%)	0.010*

*P < 0.05, **P < 0.01

Table 2 reports the comparison of 12 cord blood cytokines between the LOS and non-sepsis groups. Levels of IL-6 [127.81(399.86) vs. 31.02(127.48), *p* = 0.004] and IL-8 [130.37(202.53) vs. 52.91(101.43), *p* = 0.001] were significantly higher in the LOS group. No differences in other cytokine concentrations were observed between the groups. Analysis of quartile data for IL-6 and IL-8 revealed that patients in the highest quartile for IL-6 had an increased risk of LOS (OR = 3.554, 95% CI: 1.464–8.625, *p* = 0.005), which remained significant after adjusting for gestational age, gestational hypertension, and antenatal steroids (aOR = 3.113, 95% CI: 1.239–7.819, *p* = 0.016). Similarly, patients in the highest quartile for IL-8 also had an increased risk of LOS (OR = 4.282, 95% CI: 1.548–11.844, *p* = 0.005); however, this significance disappeared after adjustment (aOR = 2.431, 95% CI: 0.839–7.042, *p* = 0.102) (Table 3).

**Table 2:** Umbilical cord cytokine levels (pg/ml) in preterm infants with and without LOS

	LOS group [median (IQR)] #	Non-sepsis group [median (IQR)]	Total	*p*
IL-1β	2.44(3.14)	3(2.2)	3(2.2)	0.583
IL-2	2.44(1.56)	2.44(1.56)	2.44(1.56)	0.668
IL-4	2.44(0.22)	2.44(0.56)	2.44(0.56)	0.335
IL-5	3(2.64)	3(1.17)	3(1.17)	0.254
IL-6	127.81(399.86)	31.02(127.48)	33.58(140.72)	0.004**
IL-8	130.37(202.53)	52.91(101.43)	56.5(114.50)	0.001**
IL-10	7.00(22.45)	5.6(11.30)	5.88(11.71)	0.190
IL-12p70	2.44(0.12)	2.44(1.56)	2.44(0.56)	0.196
IL-17	2.44(1.17)	2.44(2.56)	2.55(2.56)	0.487
IFN-α	2.44(1.49)	2.44(2.56)	2.55(2.56)	0.239
IFN-γ	3.3(9.59)	2.605(4.24)	2.61(4.26)	0.251
TNF-α	2.44(0.64)	2.44(1.56)	2.44(1.56)	0.367

*P < 0.05, **P < 0.01 #2.44 is the minimum detection value

**Table 3:** Relationship between IL-6, IL-8 and LOS before and after adjustment

Unadjusted				Adjusted#		
	OR	95CI%	*P*	OR	95CI%	*P*
IL-6	Q1 Ref	-	-	-	-	-
Q2	1.020	0.349–2.958	0.971	1.218	0.401–3.703	0.728
Q3	1.020	0.349–2.958	0.971	1.605	0.524–4.911	0.407
Q4	3.554	1.464–8.625	0.005**	3.113	1.239–7.819	0.016*
IL-8	Q1 Ref	-	-	-	-	-
Q2	1.409	0.438–4.534	0.565	1.317	0.397–4.368	0.653
Q3	2.672	0.919–7.774	0.071	1.511	0.499–4.571	0.465
Q4	4.282	1.548–11.844	0.005**	2.431	0.839–7.042	0.102

#: Gestational age, Gestational hypertension and Antenatal steroid were included as covariates in this model

*P < 0.05, **P < 0.01

Peripheral white blood cell counts, including neutrophils, lymphocytes, and monocytes, are detailed in Table 4. Neutrophil and monocyte counts were significantly lower in the LOS group [5.08 ± 3.46 vs. 8.14 ± 4.90, *p* = 0.000; 0.98 ± 0.56 vs. 1.37 ± 0.72, *p* = 0.001, respectively]. Binary logistic regression analysis of variables with *p*-values < 0.05 showed that lower gestational age (OR = 0.748; 95% CI: 0.649–0.863; *p* = 0.000), lack of antenatal steroid administration (OR = 0.902; 95% CI: 0.127–0.912; *p* = 0.032), and low peripheral neutrophil count within 24 h (OR = 0.340; 95% CI: 0.818–0.994; *p* = 0.038) were significantly associated with the development of LOS (Table 5).

**Table 4:** Neutrophil, lymphocyte, and monocyte counts (10^9^/L) in peripheral blood of preterm infants with and without loss

	LOS group	Non-sepsis group	t	*p*
Neutrophil count	5.08 ± 3.46	8.14 ± 4.90	3.974	0.000**
Lymphocyte count	3.38 ± 1.80	3.23 ± 2.19	-0.242	0.809
Monocyte count	0.98 ± 0.56	1.37 ± 0.72	3.411	0.001**

**P < 0.01

**Table 5:** Multivariate logistic regression analysis showing the relationship between independent variables and the risk of LOS in preterm infants

	OR	95CI%	*P*
Neutrophil count	0.340	0.818–0.994	0.038*
Gestational age	0.748	0.649–0.863	0.000**
Gestational hypertension	0.666	0.313–1.416	0.291
Antenatal steroid	0.902	0.127–0.912	0.032*

*P < 0.05, **P < 0.01

## Discussion

In this retrospective cohort study, the incidence of late-onset sepsis (LOS) and its trend across different gestational ages align with previous reports [12, 13]. We found that cord blood levels of IL-6 and IL-8 were significantly higher in the LOS group compared to preterm infants without sepsis, while neutrophil and monocyte counts in peripheral blood were lower in the LOS group. Multivariable logistic regression analysis indicated that the association between IL-6 and neutrophil counts with LOS remained significant after adjusting for other confounding factors, which has not been previously reported.

During the early stages of chorioamnionitis, the maternal immune response predominates and infiltrates the membranes. As the infection or inflammation progresses, fetal leukocytes begin to infiltrate, resulting in elevated concentrations of pro-inflammatory cytokines such as IL-1β, IL-4, IL-6, and IL-8 [14]. Research has shown that neonates with severe intra-amniotic inflammation exhibit significantly elevated cord blood cytokine levels, which can persist beyond the immediate neonatal period [15, 16]. Increasing evidence suggests that intrauterine inflammation exposure during pregnancy impacts subsequent immune responses [17, 18]. However, most clinical studies linking chorioamnionitis to offspring LOS have focused solely on placental pathology without direct evidence of fetal inflammatory responses. The stage and duration of intrauterine inflammation exposure remain unclear, leading to varying conclusions. Our study utilized cord blood cytokine levels to provide precise evidence that intrauterine inflammation exposure in preterm infants alters immune status and increases the risk of LOS, with IL-6 playing a crucial role.

IL-6, a pro-inflammatory cytokine, is a hallmark of fetal inflammatory response syndrome (FIRS) [10]. Maternal infection can induce inflammation in offspring through IL-6 independently of the microbiota [19]. Additionally, IL-6 is an independent predictor of adverse neonatal outcomes, including bronchopulmonary dysplasia (BPD) and white matter injury (WMI) in preterm infants [20–22]. Our findings suggest that IL-6 may also serve as an independent predictor of LOS.

Recent interest has focused on trained immunity, where innate immune cells are “trained” by initial stimuli or infections to respond more robustly to subsequent challenges [23]. Research has demonstrated altered DNA methylation signatures in placentas associated with chorioamnionitis, consistent with the activation of innate immune responses [24]. In our study, in addition to changes in cord blood IL-6, we also found significant differences in the neutrophil and monocyte counts in preterm infants on the first day of life. And in neutrophils this difference still exists after adjusting confounders. Similar observations of neutrophil-driven inflammation have been observed in animal models. LPS-induced maternal inflammation promotes fetal leukocyte recruitment and prenatal organ infiltration in mice [25]. During the inflammatory process, IL-6 trans-signaling is involved in many processes including mononuclear cell recruitment and apoptosis of neutrophils [26]. This may lead to a decreased level of neutrophils and monocyte in peripheral blood. Emma de Jong et al. showed that chorioamnionitis-exposure is associated with reduced monocyte expression of key immune genes and global analysis of monocyte transcriptome reveals hypo-responsiveness to S. epidermidis in infants exposed to chorioamnionitis [27]. An animal study conducted by Ai Ing Lim, further showed that increasing maternal IL-6 levels during pregnancy can alter the innate immune response of offspring by changing the epigenome [19]. Our study supports the hypothesis that elevated cord blood IL-6 increases the incidence of LOS through “trained immunity”.

IL-8, a pro-inflammatory cytokine from the chemokine family, is crucial in inflammation, immune response, and tissue repair. Although the highest quartile of IL-8 was associated with increased LOS risk, this significance disappeared after adjusting for gestational age. Elevated antenatal vaginal IL-8 levels have been reported in high-risk women who delivered preterm compared to term deliveries, with IL-8 being an independent predictor of preterm birth [28]. Thus, the association between IL-8 and LOS may be primarily mediated by gestational age.

The association between early neutropenia and LOS deserves particular emphasis. Our data, supported by other studies [29, 30], reveal that even subtle neutrophil reductions - not meeting traditional neutropenia thresholds - significantly increase infection risk. Preterm infants face a dual challenge: quantitative deficits in neutrophil reserves compounded by qualitative impairments in chemotaxis, phagocytosis, and oxidative burst capacity. This functional immaturity creates a perfect storm for microbial invasion, particularly when combined with the immune-exhaustive effects of prior inflammatory exposure.

To our knowledge, this study is among the few cohort studies investigating the relationship between cord blood cytokine levels, peripheral white blood cell counts, and LOS in preterm infants. It is also relatively comprehensive in its cytokine detection. This study has several limitations. Firstly, while the inclusion of all preterm infants enhances the generalizability of our findings, the relatively small sample size of very preterm infants, who are particularly vulnerable to LOS, may limit the robustness of subgroup analyses. Secondly, we did not examine correlations between the studied markers (e.g., IL-6, neutrophil counts) and specific LOS pathogens. As certain markers may be more strongly associated with particular pathogens, this could facilitate targeted antibiotic therapy [31]. Future studies should explore these relationships to better understand the etiology of LOS. Lastly, the study did not evaluate long-term outcomes among LOS patients, limiting our ability to link early markers to subsequent neurodevelopmental or health trajectories.

Future research should investigate the clinical utility of these markers in improving morbidities, complications, and mortality rates associated with LOS.

## Conclusion

High levels of IL-6 in cord blood and low peripheral neutrophil counts on the first day of life are associated with an increased risk of late-onset sepsis in preterm infants. Prenatal exposure to inflammation may elevate the risk of late-onset sepsis in preterm infants, partially by altering immune system responses later in life.

## Data Availability

The original contributions presented in the study are included in the article/supplementary material, further inquiries can be directed to the corresponding authors.
